# Supplementary material for: Unveiling the Bioactive Potential of the Invasive Jellyfish Phyllorhiza punctata Through Integrative Transcriptomic and Proteomic Analyses
Source: Biomolecules. 2025 Aug 4;15(8):1121. doi: 10.3390/biom15081121 (PMC12383608; doi:10.3390/biom15081121)
Supplement: Supplementary file 1 [file biomolecules-15-01121-s001.zip › Suplementary Material/FileS3/PEPFOLD4/XP_065063213_12254/clusters.html]

 PEP-FOLD clusters


Download archive of all models  here   

|  |  |  |
| --- | --- | --- |
| # | file | sOPEP |
| # cluster 1 - Size: 14 |  |  |
|  | XP\_065063213\_12254-model1 | -6.90402 |
|  | XP\_065063213\_12254-model1.1 | -6.71756 |
|  | XP\_065063213\_12254-model1.2 | -6.37361 |
|  | XP\_065063213\_12254-model1.3 | -5.93728 |
|  | XP\_065063213\_12254-model1.4 | -5.38868 |
|  | XP\_065063213\_12254-model1.5 | -5.12033 |
|  | XP\_065063213\_12254-model1.6 | -4.89781 |
|  | XP\_065063213\_12254-model1.7 | -4.77125 |
|  | XP\_065063213\_12254-model1.8 | -3.77872 |
|  | XP\_065063213\_12254-model1.9 | -3.72303 |
|  | XP\_065063213\_12254-model1.10 | -3.6943 |
|  | XP\_065063213\_12254-model1.11 | -3.10642 |
|  | XP\_065063213\_12254-model1.12 | -3.10356 |
|  | XP\_065063213\_12254-model1.13 | -2.63723 |
| # cluster 2 - Size: 1 |  |  |
|  | XP\_065063213\_12254-model2 | -6.54707 |
| # cluster 3 - Size: 7 |  |  |
|  | XP\_065063213\_12254-model3 | -5.9344 |
|  | XP\_065063213\_12254-model3.1 | -5.67287 |
|  | XP\_065063213\_12254-model3.2 | -5.67287 |
|  | XP\_065063213\_12254-model3.3 | -4.9788 |
|  | XP\_065063213\_12254-model3.4 | -4.32651 |
|  | XP\_065063213\_12254-model3.5 | -4.02594 |
|  | XP\_065063213\_12254-model3.6 | -2.66259 |
| # cluster 4 - Size: 16 |  |  |
|  | XP\_065063213\_12254-model4 | -5.64753 |
|  | XP\_065063213\_12254-model4.1 | -5.05505 |
|  | XP\_065063213\_12254-model4.2 | -4.98586 |
|  | XP\_065063213\_12254-model4.3 | -4.89544 |
|  | XP\_065063213\_12254-model4.4 | -4.4964 |
|  | XP\_065063213\_12254-model4.5 | -4.28617 |
|  | XP\_065063213\_12254-model4.6 | -4.04861 |
|  | XP\_065063213\_12254-model4.7 | -3.92797 |
|  | XP\_065063213\_12254-model4.8 | -3.92299 |
|  | XP\_065063213\_12254-model4.9 | -3.91566 |
|  | XP\_065063213\_12254-model4.10 | -3.86905 |
|  | XP\_065063213\_12254-model4.11 | -3.64336 |
|  | XP\_065063213\_12254-model4.12 | -2.99307 |
|  | XP\_065063213\_12254-model4.13 | -2.92327 |
|  | XP\_065063213\_12254-model4.14 | -2.70303 |
|  | XP\_065063213\_12254-model4.15 | -2.68703 |
| # cluster 5 - Size: 2 |  |  |
|  | XP\_065063213\_12254-model5 | -3.51642 |
|  | XP\_065063213\_12254-model5.1 | -2.50584 |
| # cluster 6 - Size: 4 |  |  |
|  | XP\_065063213\_12254-model6 | -2.65677 |
|  | XP\_065063213\_12254-model6.1 | -2.4043 |
|  | XP\_065063213\_12254-model6.2 | -2.30774 |
|  | XP\_065063213\_12254-model6.3 | -2.28714 |
| # cluster 7 - Size: 6 |  |  |
|  | XP\_065063213\_12254-model7 | -2.64639 |
|  | XP\_065063213\_12254-model7.1 | -2.44156 |
|  | XP\_065063213\_12254-model7.2 | -2.42917 |
|  | XP\_065063213\_12254-model7.3 | -2.41137 |
|  | XP\_065063213\_12254-model7.4 | -2.36965 |
|  | XP\_065063213\_12254-model7.5 | -2.29056 |
| # cluster 8 - Size: 2 |  |  |
|  | XP\_065063213\_12254-model8 | -2.62246 |
|  | XP\_065063213\_12254-model8.1 | -2.45563 |
| # cluster 9 - Size: 5 |  |  |
|  | XP\_065063213\_12254-model9 | -2.61863 |
|  | XP\_065063213\_12254-model9.1 | -2.51432 |
|  | XP\_065063213\_12254-model9.2 | -2.48021 |
|  | XP\_065063213\_12254-model9.3 | -2.47992 |
|  | XP\_065063213\_12254-model9.4 | -2.26919 |
| # cluster 10 - Size: 1 |  |  |
|  | XP\_065063213\_12254-model10 | -2.58874 |
| # cluster 11 - Size: 3 |  |  |
|  | XP\_065063213\_12254-model11 | -2.57174 |
|  | XP\_065063213\_12254-model11.1 | -2.51846 |
|  | XP\_065063213\_12254-model11.2 | -2.49827 |
| # cluster 12 - Size: 3 |  |  |
|  | XP\_065063213\_12254-model12 | -2.52453 |
|  | XP\_065063213\_12254-model12.1 | -2.40866 |
|  | XP\_065063213\_12254-model12.2 | -2.39181 |
| # cluster 13 - Size: 4 |  |  |
|  | XP\_065063213\_12254-model13 | -2.52296 |
|  | XP\_065063213\_12254-model13.1 | -2.46912 |
|  | XP\_065063213\_12254-model13.2 | -2.45311 |
|  | XP\_065063213\_12254-model13.3 | -2.44386 |
| # cluster 14 - Size: 8 |  |  |
|  | XP\_065063213\_12254-model14 | -2.52231 |
|  | XP\_065063213\_12254-model14.1 | -2.50003 |
|  | XP\_065063213\_12254-model14.2 | -2.47472 |
|  | XP\_065063213\_12254-model14.3 | -2.46667 |
|  | XP\_065063213\_12254-model14.4 | -2.45444 |
|  | XP\_065063213\_12254-model14.5 | -2.44318 |
|  | XP\_065063213\_12254-model14.6 | -2.42517 |
|  | XP\_065063213\_12254-model14.7 | -2.36565 |
| # cluster 15 - Size: 5 |  |  |
|  | XP\_065063213\_12254-model15 | -2.50259 |
|  | XP\_065063213\_12254-model15.1 | -2.49613 |
|  | XP\_065063213\_12254-model15.2 | -2.444 |
|  | XP\_065063213\_12254-model15.3 | -2.43404 |
|  | XP\_065063213\_12254-model15.4 | -2.36146 |
| # cluster 16 - Size: 5 |  |  |
|  | XP\_065063213\_12254-model16 | -2.48816 |
|  | XP\_065063213\_12254-model16.1 | -2.46844 |
|  | XP\_065063213\_12254-model16.2 | -2.43755 |
|  | XP\_065063213\_12254-model16.3 | -2.36728 |
|  | XP\_065063213\_12254-model16.4 | -2.35011 |
| # cluster 17 - Size: 5 |  |  |
|  | XP\_065063213\_12254-model17 | -2.48403 |
|  | XP\_065063213\_12254-model17.1 | -2.44498 |
|  | XP\_065063213\_12254-model17.2 | -2.3935 |
|  | XP\_065063213\_12254-model17.3 | -2.31721 |
|  | XP\_065063213\_12254-model17.4 | -2.29978 |
| # cluster 18 - Size: 1 |  |  |
|  | XP\_065063213\_12254-model18 | -2.48161 |
| # cluster 19 - Size: 1 |  |  |
|  | XP\_065063213\_12254-model19 | -2.4723 |
| # cluster 20 - Size: 1 |  |  |
|  | XP\_065063213\_12254-model20 | -2.46601 |
| # cluster 21 - Size: 1 |  |  |
|  | XP\_065063213\_12254-model21 | -2.44554 |
| # cluster 22 - Size: 1 |  |  |
|  | XP\_065063213\_12254-model22 | -2.43343 |
| # cluster 23 - Size: 1 |  |  |
|  | XP\_065063213\_12254-model23 | -2.426 |
| # cluster 24 - Size: 1 |  |  |
|  | XP\_065063213\_12254-model24 | -2.38536 |
| # cluster 25 - Size: 1 |  |  |
|  | XP\_065063213\_12254-model25 | -2.36138 |
| # cluster 26 - Size: 1 |  |  |
|  | XP\_065063213\_12254-model26 | -2.34392 |
|  |  |  |
